# Urdu version of the neck disability index: a reliability and validity study

**DOI:** 10.1186/s12891-017-1469-5

**Published:** 2017-04-08

**Authors:** Muhammad Nazim Farooq, Mohammad A. Mohseni-Bandpei, Syed Amir Gilani, Ambreen Hafeez

**Affiliations:** 1 University Institute of Physical Therapy, Faculty of Allied Health Sciences, University of Lahore, Lahore, Pakistan; 2 Islamabad College of Physiotherapy, Margalla Institute of Health Sciences, Quaid-e-Azam Avenue, Gulrez III, Rawalpindi, Pakistan; 3 Pediatric Neurorehabilitation Research Center, University of Social Welfare and Rehabilitation Sciences, Tehran, Iran; 4 Dean Faculty of Allied Health Sciences, Director; Directorate of International Linkages, University of Lahore, Lahore, Pakistan; 5 Physiotherapy Department, KRL General Hospital, Kahuta, Distt., Rawalpindi, Pakistan

**Keywords:** Disability, Neck, Reliability, Translations, Validity

## Abstract

**Background:**

Despite the wide use of the neck disability index (NDI) for assessing disability in patients with neck pain, the NDI has not yet been translated and validated in Urdu. The first purpose of the present study was to translate and cross-culturally adapt the NDI into the Urdu language (NDI-U). The second purpose was to investigate the reliability, validity and responsiveness of the NDI-U in Urdu-speaking patients experiencing chronic mechanical neck pain (CMNP).

**Methods:**

Translation and cross-cultural adaptation of the original version of the NDI were carried out using previously described procedures. Seventy-six patients with CMNP and thirty healthy participants were recruited for the study. NDI-U and visual analogue scales for pain intensity (VAS_pain_) and disability (VAS_disability_) were administered to all the participants at baseline and to the patients 3 weeks after receiving physiotherapy intervention. The global rating of change scale (GROC) was also administered at this time. Test-retest reliability and internal consistency were carried out on forty-six randomly selected patients two days after they completed the NDI-U. The NDI-U was evaluated for factor analysis, content validity, construct validity (discriminative and convergent validity) and responsiveness.

**Results:**

An intra-class correlation coefficient (ICC_2,1_) revealed excellent test-retest reliability for all items (ICC_2,1_ = 0.86–0.98) and total scores (ICC_2,1_ = 0.99) of the NDI-U. The NDI-U was found internally consistent with a Cronbach’s alpha of 0.90 and a fair to good correlation between single items and the NDI-U total scores (*r* = 0.34 to 0.89). Factor analysis of the NDI-U produced two factors explaining 66.71% of the variance. Content validity was good, as no floor or ceiling effects were detected for the NDI-U total score. To determine discriminative validity, an independent *t*-test revealed a significant difference in the NDI-U total scores between the patients and healthy controls (*P* < 0.001). For convergent validity, Pearson’s correlation coefficient showed a strong correlation between NDI-U and VAS_disability_ (*r* = 0.83, *P* < 0.001) and a moderate correlation between NDI-U and VAS_pain_ (*r* = 0.62, *P* < 0.001). To measure responsiveness, an independent *t*-test showed a significant difference in the NDI-U change scores between the stable and the improved groups (*P* < 0.001). Furthermore, moderate correlations were found between the NDI-U change scores and the GROC (*r* = 0.50, *P* < 0.001), VAS_disability_ change scores (*r* = 0.58, *P* < 0.001) and VAS_pain_ change scores (*r* = 0.55, *P* < 0.001).

**Conclusion:**

The results showed that the NDI-U is a reliable, valid and responsive questionnaire to measure disability in Urdu-speaking patients with CMNP.

**Electronic supplementary material:**

The online version of this article (doi:10.1186/s12891-017-1469-5) contains supplementary material, which is available to authorized users.

## Background

Neck pain is a major health problem with an annual prevalence ranging from 4.8 to 79.5% in the general population [1, 2]. In 50%–80% of patients with neck pain, the symptoms do not resolve completely [3]. Neck pain may result in disability that significantly affects an individual’s activities and reduces their ability to perform activities of daily living [4]. Therefore, it is essential to use a reliable and valid measurement tool to determine a patient’s perception of disability and to assess treatment outcomes in patients with neck pain [5].

Self-reported generic and region-specific questionnaires are frequently used to measure disability in patients with neck pain [6, 7]. The neck disability index (NDI) is one of the most commonly used questionnaires to measure neck pain and disability [8]. One study reported that the NDI is a multidimensional construct that measures a broader concept than disability [9]. Nonetheless, the original NDI developed by Vernon and Mior [10] is a much more reliable and validated measure of neck pain and disability, compared to other questionnaires [6]. The NDI has stable psychometric properties confirmed by different studies [11–15]. The NDI has been translated and validated in several languages [11–29], providing a standard measure to be used in clinical practices and research studies while allowing clinicians and researchers to share knowledge, study results of interventions, and compare results across different populations [6, 16].

Many studies adapt previously recognized and frequently used assessment tools instead of developing a new questionnaire [30, 31]. The reliability and validity of the Urdu version of the neck disability index (NDI-U) has not been studied. The aim of the present study was to translate and culturally adapt the NDI to the Urdu language according to established procedures and to test the psychometric properties of the translated version in Urdu-speaking patients with chronic mechanical neck pain (CMNP).

## Methods

### Translation and cultural adaptation

The translation and cultural adaptation processes were started after obtaining approval from the developer of the original NDI. These processes were performed according to the guidelines previously described [31] and to the COSMIN (COnsensus-based Standards for the selection of health status Measurement INstruments) criteria [32]. The entire process consisted of five steps.

#### Step I

Two native Urdu-speaking translators who were also fluent in English independently translated the NDI from English into Urdu. One of the translators was an English linguistic teacher, and the second was a physiotherapist. Both translators were instructed to aim for conceptual rather than literal translation. They both provided written reports.

#### Step II

The original translators and one of the authors produced a consensus version by synthesizing the results of both translated versions and discussing disagreements.

#### Step III

The agreed upon Urdu version was translated back into English by two professional translators who were blinded to the original version. Both translators were not aware of the questionnaire concept.

#### Step IV

An expert committee including translators, researchers, a healthcare professional and a methodologist developed a pre-final version by reviewing all the translations, the consensus version, and the original questionnaire. The entire procedure was recorded.

#### Step V

The pre-final version of the NDI-U was tested on 30 patients with neck pain to test for face validity. The patients were requested to complete the questionnaire. Afterwards, all the items of the questionnaire were discussed with the patients one by one. We asked patients to describe what they understand about each question and to provide their impressions of the relevance of the items to their situation and their ability to complete the questionnaire on their own. Patients were also encouraged to note any problems with the wording, instructions or layout of the questionnaire. All findings from this phase of the adaptation process were evaluated by the expert committee, and the final NDI-U was then developed following consensus (Additional file 1).

### Instruments

#### Neck Disability Index (NDI)

The NDI was derived from the Oswestry Disability Index [33], and it consists of ten items related to pain intensity, headache, concentration and different physical activities (lifting, personal care, recreation, work, driving, reading and sleeping) with six possible responses per item [10]. The score of each item ranges from 0 to 5 [10]. The highest total possible score is 50, and this score is converted to a percentage. Higher scores represent higher levels of disability [10]. The NDI has been shown to be a valid and reliable questionnaire for patients with neck pain [10, 34, 35].

#### Visual analogue scale for pain (VAS_pain_)

The VAS_pain_ consists of a 100 mm horizontal line with the words “no pain” and “worst possible pain” at the line’s ends [36, 37]. Patients were asked to quantify their neck pain by drawing a vertical mark on the area of the horizontal line that best represented their pain level during the preceding 24 h. The VAS_pain_ has been shown to be a reliable and valid tool to measure pain intensity [36–39].

#### Visual analogue scale for disability (VAS_disability_)

The VAS_disability_ also consists of a 100 mm horizontal line with the descriptors “no restriction (0)” and “worst possible restriction (100)” at the line’s ends. Patients were asked to quantify how much their neck pain restricts their daily activities by drawing a vertical mark on the area of the horizontal line that best represented their degree of restriction. The VAS_disability_ has been shown to have good reliability in patients with chronic musculoskeletal pain [40].

#### Global Rating of Change (GROC)

The GROC is a 15 point scale that is used to assess a patient’s self-perception of pain deterioration or improvement over time [41]. Patients were requested to rate the overall condition of their neck from −7 (“a very great deal worse”) to +7 (“a very great deal better”) since the start of treatment. The GROC has been shown to be a validated measure and is widely used as a reference standard to test other instruments [21, 29, 41–43]. Unlike other questionnaires used to assess health status, the GROC scale is simple, quick, easy to use and requires no special training or skills to administer [41].

### Psychometric testing

Psychometric testing of the NDI-U was performed according to COSMIN guidelines [32].

#### Participants

Patients with CMNP were recruited from two hospitals located in Rawalpindi and Islamabad, Pakistan, over a period of 12 months. Neck pain was defined as chronic if the duration of the symptoms was more than three months [44]. Both male and female patients between 18 and 65 years of age who were able to read Urdu were included in the study. Patients were excluded if they had any of the following co-morbid diagnoses: inflammatory diseases, current infection, tumours, history of fracture and surgery on the cervical spine, severe cervical myelopathy or radiculopathy, pregnancy or extensive psychiatric disorders. Moreover, 30 healthy volunteers who had no history of pain or neck pathology who were between 19 and 26 years of age were also recruited from the staff and students of the Margalla Institute of Health Sciences Rawalpindi.

The study was approved by the Institutional Review Board of the University of Lahore, Lahore, Pakistan. All the participants provided informed written consent. The screening of the participants was carried out by physiotherapists with more than ten years of clinical experience.

#### Procedure

During the first visit, self-report measures for the NDI-U, VAS_pain_ and VAS_disability_ were completed by the healthy participants and patients with neck pain. Weight, height and other demographic details were also recorded. After 48 h, 46 randomly selected patients completed the NDI-U again. These patients received 9 sessions (3/week) of physiotherapy treatment with each session lasting for 30 min. These were provided by physiotherapists with clinical experience of more than twelve years. After 3 weeks of physiotherapy, patients again completed the NDI-U, VAS_pain_ and VAS_disability_. Additionally, patients also filled out the GROC scale at this time.

#### Strategies for missing items on the NDI

One fundamental problem with the NDI is that a few items (especially driving and reading) are frequently omitted by some patients [21]. Different strategies can be used to handle these missing values [8]. Questionnaires with 1–2 missing items were included in the present study. The patient’s total score was divided by 9 or 8 (for 1 or 2 missing items, respectively), and this average score value was used as a score for the missing item [8]. Any questionnaire with more than two unanswered items was not accepted and removed from the study [8].

Similar to previous studies, all patients were asked to explain why a question was not answered in a space provided at the end of the NDI-U [14, 21]. Furthermore, for all measurements, the same instructions that were printed on the questionnaires were also given verbally to all patients by the research assistant.

### Data analyses

All analyses were carried out using IBM SPSS 21 (IBM Corp., Armonk, NY) statistical software. The significance level was set at 0.05. Participants’ characteristics were compared using descriptive statistics.

#### Reliability

Reliability is defined as “the extent to which the measurement of a variable is free from measurement error” [45]. In the present study, the reliability of the NDI-U was determined by assessing test-retest reliability across repeated measures, internal consistency and measurement errors [45]. We expected that the test-retest coefficient would be > 0.80, and we set the value of Cronbach’s alpha of the NDI-U to be ≥ 0.70 [10, 12, 14, 16, 19, 25, 29, 35, 46, 47]. A fair to moderate correlation (0.25 ≤ r < 0.75) between single items and the total score was expected [10, 15, 25, 35]. Reliability was tested in 46 randomly selected patients from the total sample who completed the NDI-U. These individuals were re-tested after two days in the same way that they were tested the first time. During this period, patients were not provided with any treatment. The sample size was set based on previously developed methods [48] using a power calculation to determine the required sample size for a reliable study.

Test-retest reliability was determined using an intra-class correlation coefficient (ICC_2,1_) and 95% confidence intervals (CIs) [14, 15, 32]. ICC values of ≥ 0.75 are considered to represent studies with excellent reliability [49, 50]. Cronbach’s alpha was calculated to determine the internal consistency of the NDI-U [32, 51]. Alpha values between 0.70 and 0.95 are considered to be acceptable [52]. The strength of the relationship between single items and total scores of the NDI-U was assessed by computing Spearman’s correlation coefficients between each item and the total score minus the score of the item being investigated [25]. Measurement error was determined by calculating the standard error of measurement (SEM) and the smallest detectable change (SDC) [32]. The SEM represents the standard deviation (SD) of repeated measures in the same patient. It was computed using the formula SD × √ (1 – ICC) [53]. The SDC is the smallest change that showed the change observed is real and not due to measurement error. The SDC was calculated as 1.96 × √2 × SEM [52, 53].

#### Factor analysis

Factor analysis is frequently used to determine if items of an instrument form one or more than one dimension [54, 55]. Factor analysis was performed using the principal component factor analysis with varimax rotation. Clusters of items were identified using eigenvalues > 1 [29]. Factor loadings ≥ 0.4 was considered adequate [29, 54]. Keiser-Meyer-Olkin (KMO) test and Bartlett’s test of sphericity were used to determine if correlations were sufficiently large to perform a factor analysis [56]. Given earlier studies showing a one-factor or two-factor structure of the NDI in other translations, an a priori assumption about the underlying factor structure of the NDI-U was not made.

#### Content validity

Content validity is the degree to which the content of an instrument has an adequate reflection of the construct being measured [45]. Content validity was assessed by determining the completeness of item responses and the size of floor and ceiling effects [25, 57]. We expected that there would be less than 5% missing items for the cumulative responses of all the patients and that there would be no floor and ceiling effects [11, 14, 16, 25, 29, 46, 47]. Floor and ceiling effects were considered to be present if > 15% of the respondents achieved the lowest or highest possible total score [8].

#### Construct validity

Construct validity was assessed by determining the differences in the NDI-U total scores between patients and healthy controls (discriminative validity) with an independent *t*-test. We predicted that there would be a significant difference in total scores between these two groups [14]. Construct validity was also assessed by measuring the correlation between NDI-U and VAS_disability_ and VAS_pain_ (convergent validity) using Pearson’s correlation coefficients [25]. A moderate correlation between NDI-U and VAS_disability_ [11, 25, 46, 47] and a fair to moderate correlation between NDI-U and VAS_pain_ [11, 17, 19, 25, 35, 46, 47] were expected. The validity was considered good when at least 75% of the results matched the hypotheses [52].

#### Responsiveness

Responsiveness is defined as “the ability of an instrument to detect change over time in the construct to be measured” [45]. After three weeks of treatment, patients were divided into an improved group (GROC ≥ 3 (somewhat better)) and a stable group (GROC < 3 to > −3) [58]. The change in GROC scores between −3 and 3 has been described as minimal to no change [59]. Responsiveness was analysed by comparing the NDI-U change scores between these two groups with an independent *t*-test [29, 60]. We predicted that there would be a significant difference in the NDI-U change scores between the improved and stable groups [29, 60]. We also assessed responsiveness by correlating the NDI-U change scores to the GROC [21, 29] and by correlating the change scores of the NDI-U with the change scores of the VAS_pain_ and VAS_disability_ [57]. Pearson’s correlation coefficients were used to quantify these relationships. Moderate correlations were expected between the NDI-U change scores and the GROC, VAS_disability_ and VAS_pain_ change scores.

Portney and Watkins [61] criteria were used to interpret the correlations as follows: *r* < 0.25 indicates no or little correlation, 0.25 ≤ r < 0.50 indicates fair correlation, 0.50 ≤ r < 0.75 indicates moderate correlation, and 0.75 ≤ r ≤ 1 indicates good correlation.

## Results

### Translation and cultural adaptation

There were 13 patients who did not know how to drive a car, so they did not respond to item 8, which was related to driving. One patient did not answer item 4 related to reading, stating that he did not want to give an answer based on an assumption, as the item was not related to his life. It was decided not to change these sections since these problems could be overcome by any type of modification.

After thoroughly discussing replacing the word “pain” with “neck pain” for the items related to lifting, personal care and pain intensity and adding the option “never done” for the item related to driving, modifications performed in other translations [21, 29, 46], we decided to avoid such changes so as to be as close to the original version as possible. The patients’ general impression of the NDI-U was that both the instructions and items of the questionnaire were easy to understand and easy to complete. Furthermore, patients stated that all the included items were relevant to their underlying pain condition. Therefore, no major change was made to NDI-U after performing the pre-test.

### Psychometric testing

**Fig. 1:**
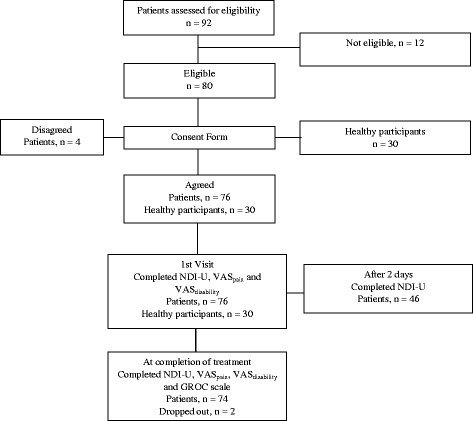
Flow chart of participants’ recruitment and measurements. *NDI-U* Urdu version of the neck disability index, *VAS* Visual analogue scale, *GROC* Global rating of change

Ninety-two patients with chronic neck pain were assessed for eligibility. Twelve patients did not meet the inclusion criteria and were excluded from the study (Fig. 1). Four patients declined to participate. The eligible patients included 30 males and 46 females. Two patients dropped out during the treatment and therefore did not complete the NDI-U, VAS_pain_, VAS_disability_ and GROC scale upon the completion of treatment. The data of these patients were not included in the follow-up analysis. The healthy participants were sex-matched to the patients. The demographic and clinical characteristics of the participants are shown in Table 1.

**Table 1:** Participant characteristics

Variables		Patient Group (*n* = 76)	Healthy Group (*n* = 30)
		Mean ± SD	Mean ± SD
		N/%	N/%
Age, years		43.37 ± 11.94	21.27 ± 1.70
Sex, female		46/60.5	17/56.7
BMI		26.28 ± 4.62	23.22 ± 3.03
VAS_pain_ (cm), range 0-10		5.02 ± 2.12	0
Duration of neck pain in months		10.50 (3–24)^a^	N/A
NDI-U, range 0–50		15.68 ± 8.52	0
VAS_disability_ (cm), range 0–10		2.84 ± 1.88	0
Work status	Employed	43/56.6	1/3.3
	Un-employed	33/43.4	29/96.7
Education	Primary	7/9.2	-
	Matric	5/6.6	-
	Intermediate	20/26.3	29/96.7
	Graduation	28/36.8	1/3.3
	Post-graduation	16/21.1	-

*BMI* Body mass index, *NDI-U* Urdu version of the neck disability index, *VAS* Visual analogue scale

^a^Median value with percentiles (P25–P75)

#### Test-retest reliability and internal consistency

The mean and standard deviation for scores of all the items, the total scores, and the reliability results of the NDI-U are shown in Table 2. The results demonstrated excellent test-retest reliability for all the items (ICC_2,1_ = 0.86–0.98) and total scores (ICC_2,1_ = 0.99) of the NDI-U. An excellent internal consistency was demonstrated with Cronbach’s alpha of 0.90. A fair to good correlation was found between single items and total scores of the NDI-U with Spearman’s correlation coefficients of 0.34 to 0.89, confirming that the NDI-U is internally consistent. The SEM and SDC for NDI-U total scores were 0.84 and 2.33, respectively.

**Table 2:** Test-retest reliability, measurement errors, Cronbach’s alpha and item-total correlation values for NDI-U (*n* = 46)

NDI-U Score	1^st^ Measurement	2^nd^ Measurement	ICC (95% CI)	SEM	SDC	Cronbach’s alpha	Item-total correlation
	Mean ± SD	Mean ± SD					
Question 1	2.09 ± 0.91	1.85 ± 0.87	0.86 (0.75–0.92)	0.32	0.89	NA	0.69
Question 2	0.91 ± 1.07	0.91 ± 1.05	0.98 (0.96–0.99)	0.15	0.42	NA	0.67
Question 3	2 ± 1.38	1.91 ± 1.31	0.96 (0.94–0.98)	0.27	0.75	NA	0.34
Question 4	1.78 ± 1.13	1.70 ± 1.13	0.97 (0.94–0.98)	0.19	0.53	NA	0.73
Question 5	1.50 ± 1.34	1.43 ± 1.31	0.98 (0.97–0.99)	0.19	0.53	NA	0.49
Question 6	1.39 ± 1.24	1.41 ± 1.20	0.98 (0.96–0.99)	0.17	0.47	NA	0.84
Question 7	1.30 ± 1.01	1.37 ± 1.06	0.95 (0.91–0.97)	0.23	0.64	NA	0.67
Question 8	1.45 ± 1.08	1.46 ± 1.07	0.93 (0.88–0.96)	0.28	0.78	NA	0.81
Question 9	1.26 ± 1.18	1.20 ± 1.09	0.96 (0.93–0.98)	0.22	0.61	NA	0.61
Question 10	1.50 ± 1.28	1.46 ± 1.33	0.97 (0.95–0.99)	0.22	0.61	NA	0.89
Total (0–50)	15.18 ± 8.58	14.70 ± 8.37	0.99 (0.98–0.99)	0.84	2.33	0.90	NA

*NDI-U* Urdu version of the neck disability index, *ICC* Intra-class correlation coefficient, *CI* confidence interval, *SEM* Standard error of measurement, *SDC* Smallest detectable change, *NA* not applicable

#### Factor analysis

**Fig. 2:**
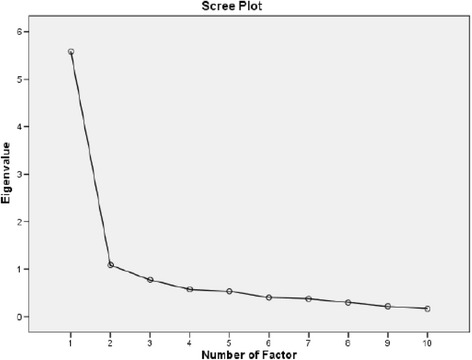
Scree plot showing the two-factor structure of the NDI-U

The results of a KMO measure of sampling adequacy and Bartlett’s test of sphericity found that the KMO value was satisfactorily high (0.90) and that the Bartlett’s test was significant (*P* < 0.001). Based on eigenvalues > 1, a two-factor structure was demonstrated. The eigenvalue of the first factor was 5.59, which explained 36.16% of the variance. The second factor had an eigenvalue of 1.08, which explained an additional 30.55% of the variance. The total variance explained by the two factors was 66.71%. A Scree Plot (Fig. 2) also supported the presence of a two-factor structure because the plotted line straightens out after the first two factors. Factor loading for all items is shown in Table 3.

**Table 3:** Factor loading values

Items	Factors 1	Factors 2
Pain Intensity	0.309	0.614^a^
Personal Care	0.744^a^	0.306
Lifting	−0.087	0.856^a^
Reading	0.536	0.616^a^
Headache	0.768^a^	0.112
Concentration	0.657^a^	0.588
Work	0.455	0.667^a^
Driving	0.487	0.628^a^
Sleeping	0.808^a^	0.127
Recreation	0.744^a^	0.508

^a^Factor loading ≥ 0.4

#### Content validity

Mean scores of individual items ranged from 1.21 to 2.16 (Table 4). Descriptive statistics showed 27 patients with 1 missing item (item 8) and 5 patients with 2 missing items (item 4 & 8). Missing responses to items represented less than 5% of the total 760 NDI-U items. No floor and ceiling effects were detected for the NDI-U total score, as no patient achieved the lowest or highest possible total scores. However, the items related to personal care, headache, concentration, work, and sleeping had floor effects with 31.5, 30.3, 25, 17.1, and 35.5% of the patients scoring the lowest possible value, respectively. There were no ceiling effects for the individual items.

**Table 4:** Descriptive data of NDI-U items and distribution of responses (*n* = 76)

NDI-U	Mean	SD	Lowest Score	Highest Score	No. of patients with missing response to an item
Pain Intensity	2.16	0.88	1	4	0
Personal Care	1.21	1.11	0	5	0
Lifting	1.79	1.31	0	5	0
Reading	1.76	1.10	0	5	5
Headache	1.49	1.32	0	4	0
Concentration	1.43	1.17	0	5	0
Work	1.47	1.04	0	4	0
Driving	1.46	1.04	0	5	32
Sleeping	1.29	1.25	0	5	0
Recreation	1.58	1.25	0	5	0
Total Score (0–50)	15.68	8.52	2	40	NA

*NDI-U* Urdu version of the neck disability index

#### Construct validity

Results showed a significant difference in the NDI-U total scores between patients and healthy controls (*P* < 0.001). Subgroup analyses between patients (*n* = 23) and healthy controls (*n* = 30) of similar age groups also showed significant differences in the total scores (*P* < 0.001). A good correlation was found between NDI-U and VAS_disability_ (Pearson’s correlation coefficient = 0.83, *P* < 0.001), and a moderate correlation was observed between NDI-U and VAS_pain_ (Pearson’s correlation coefficient = 0.62, *P* < 0.001). The results are shown in Table 5.

**Table 5:** Results for construct validity testing

Differences in NDI-U total scores	Mean ± SD	*P* value
Patients	15.68 ± 8.52	<0.001
Healthy participants	0.00	
Pearson’s correlation	R	*P* value
Between NDI-U and VAS_disability_	0.83	<0.001
Between NDI-U and VAS_pain_	0.62	<0.001

*NDI-U* Urdu version of the neck disability index, *VAS* Visual analogue scale

#### Responsiveness

An independent *t*-test found a statistically significant difference in the NDI-U change scores between the two groups (9.02 ± 6.78 in the improved group, *n* = 49; 2.67 ± 4.26 in the stable group, *n* = 25; *P* < 0.001). A moderate correlation was found between the NDI-U change scores and GROC values (Pearson’s correlation coefficient = 0.50, *P* < 0.001). A moderate correlation was also found between NDI-U and VAS_disability_ change scores (Pearson’s correlation coefficient = 0.58, *P* < 0.001) and between NDI-U and VAS_pain_ change scores (Pearson’s correlation coefficient = 0.55, *P* < 0.001).

## Discussion

As far as we know, this is the first study that translated and cross culturally adapted the NDI into Urdu and tested the reliability, validity and responsiveness of the NDI-U. The psychometric properties of the NDI-U were tested using pre-defined hypotheses. The results indicated that NDI-U has good reliability, validity and responsiveness.

Studying the adaptation process showed that the NDI-U was successfully developed according to established guidelines. The difficulties encountered during the adaptation process were handled by consensus decisions and the use of careful wording. The NDI-U was found to be simple and easy to use in clinical settings.

In the present study, there were more females (60.5%) than males (39.5%). This is comparable to earlier studies that have also recruited more females (52–78%) [14–16, 20, 21, 25, 35, 62–65] but in contrast to the Arabic version of the NDI that included more males (69.2%) than females (30.8%) [29]. In current study, the patients had mean age of 43 years, which is comparable to previous studies (35–47 years) [14, 15, 20, 25, 29, 63, 65]. However, in some other studies, the mean age of the patients was higher (50–62 year) [21, 35, 64].

An excellent internal consistency was demonstrated by a Cronbach’s alpha value of 0.90, which is well in the range of the findings of earlier studies (0.74–0.96) [10, 12, 14, 19, 21–23, 25, 28, 29, 35, 60, 64]. The variations in the correlations between single items and total scores (0.34 to 0.89) in the present study were comparable to the results of other studies (0.40 to 0.84) [10, 25, 35]. The present study found excellent test-retest reliability, comparable to the original study and other translations [10–12, 14, 17, 19, 21–23, 28, 29, 35, 60]. However, the test-retest reliability is higher compared to the German (0.81), Dutch (0.84), Italian (0.84) and Thai (0.85) versions of the NDI [26, 27, 64, 65]. Cleland et al. [66] found a very low ICC (0.50). Similarly, Cook et al. [12] found an ICC value of 0.48 upon retest. In another study conducted by Vos et al. [13], a very low ICC (0.53) was measured in the personal care item. These variations in the test-retest results may be due to the use of different intervals to determine test-retest reliability. To avoid major changes in the patients’ conditions, an interval of 2–3 days was recommended by Dawson et al. [67]. On the other hand, Deyo et al. [68] and Terwee et al. [52] recommended using a 1–2 week gap between testing and retesting to avoid memory effects. In the present study, a two-day interval was used to ensure that minimal changes in the patients’ conditions took place; the results obtained were similar to those of other studies that also used short test-retest intervals [11, 19, 46, 62, 69].

Based on the results of the current study, a change of at least 3 points on the NDI-U (0–50 scale) is required to label the change as a “real change”. This result is well within the range of findings observed in other studies (2–8 points on a 0–50 scale) [14, 21, 27, 65]. Young et al. [70] reported a SDC score of 13.4 points, but this study was performed on patients with cervical radiculopathy. In a systematic review performed by MacDermid et al. [71], the SDC was reported to be approximately 5 points (0–50 scale) for uncomplicated neck pain and approximately 10 points (0–50 scale) for cervical radiculopathy.

Many studies have analysed the factor analysis of the NDI and other translations. Some studies found a one-factor structure [12, 15, 21–23, 27, 34, 64, 72], and others found a two-factor structure [11, 14, 20, 26, 29, 60]. A two-factor structure was found in the present study, explaining 66.71% of the variance. This result is comparable to what was observed with the Japanese [60], Arabic [29], and German [14] versions, where a two-factor structure explained 61.8%, 67.58%, and 67% of the variances, respectively. However, our results of 66.71% of the variance being explained by a two-factor structure is higher than values of other versions (54–56%) [11, 20, 26]. The structure of the NDI-U is similar to those of other adaptations, with one factor related to “cognitive functioning” (items 2, 5, 6, 9, 10) and the other factor related to “pain and functional disability” (items 1, 3, 4, 7, 8). The association of the pain item with function agrees with results of the German version [14] but disagrees with results of the Arabic and original versions [20, 29]. Furthermore, the association of the driving item to “functional disability” agrees with the findings of the Catalan version [20] but disagrees with the Arabic and German versions [14, 29]. Although items 4, 7 and 8 are loaded with both factors, they are loaded more heavily with the factor labelled as “pain and functional disability”. There are some discrepancies in the factor structure of the current study compared with other studies. However, the assessment of factorial structure can be influenced by cultural differences [72].

The present study had 32 patients (42.10%) who did not complete item 8 (driving). These results are comparable with the Japanese and Greek versions where 38.2% and 44.6% of the patients, respectively, did not answer this item [21, 60]. In contrast, other studies reported less patients (2.2% to 30.76%) who did not answer item 8 [11, 15, 17, 27, 29]. One explanation to these differences may be the reason provided by our patients in that that they do not know how to drive. Thus, we assumed that the patients’ lack of response to this item was not secondary to a problem in translation; as such, we did not feel it was necessary to make any changes to this section.

There were also 5 patients (6.58%) who did not complete item 4 (reading). This result was slightly lower than that reported by Trouli et al. (9.2%) [21]. The patients who missed this item stated that they did not want to answer, as reading was not relevant to their lives.

The present study did not find any floor or ceiling effects for the NDI-U total scores. However, floor effects were observed for individual items (items 2, 5, 6, 7, 9). These results were comparable to those of the Finish (2 items) [23], Korean (3 items) [16], and Dutch (2 items) versions [25] of the NDI that have reported floor effects for individual items.

Criterion validity of the NDI-U was not analysed due to the unavailability of a gold standard for health-related questionnaires [57]. The NDI-U was found to have good construct validity. Indeed, the translated version detected significant differences in the NDI-U total scores between the patients and the healthy controls, consistent with the German version of the NDI [14]. Furthermore, the NDI-U showed positive correlations between total scores and either VAS_pain_ or VAS_disability_, consistent with previous studies [22, 25, 64]. The effect size of the correlation between NDI-U and VAS_disability_ was good (*r* = 0.83) in the present study but only moderate (*r* = 0.52) in the Dutch version of the NDI [25]. The correlation between NDI-U and VAS_pain_ (*r* = 0.58) was similar to the findings of the Iranian, Spanish, Turkish and German versions (*r* = 0.51–0.71) [14, 17, 19, 22] but higher than other versions (*r* = 0.22–0.43) [25, 64].

Regarding responsiveness, the NDI is considered to be a suitable test to detect changes over time. The NDI is frequently used in patients with neck pain to evaluate the effectiveness of treatment strategies [73]. The present study found significant differences between the stable and improved groups in their NDI-U scores, similar to previous studies [29, 60, 64]. Furthermore, a significant correlation was observed between NDI-U change scores and GROC values, which agrees with the results of the earlier studies [21, 29]. The strength of the correlation was moderate in the present study, poor in the Geek version [21], and good in the Arabic version [29]. The instrument showed positive moderate correlations between NDI-U change scores and VAS_pain_ and VAS_disability_ change scores.

### Limitations

First, a short interval was used to ensure patient conditions remained as stable as possible to determine test-retest reliability. Therefore, memory effects on our results cannot be completely ruled out. Second, our sample mainly included patients with mild to moderate disability from CMNP. Therefore, it may not be appropriate to extrapolate our results to patients with severe or (sub)acute disability or to patients having neck pain secondary to non-mechanical causes. Third, data were mainly collected from patients attending outpatient physiotherapy clinics. Therefore, the sample may not be a true representation of the general population experiencing neck pain. Consequently, the results cannot be generalized to inpatients. Finally, healthy controls were not age-matched to the patients. The authors believe that the generalizability of the results to the general population should not be affected, as the subgroup analysis between patients and healthy controls of a similar age group also found a significant difference in total scores between the two groups.

### Strengths

The strength of this study is that the psychometric properties of the NDI-U were tested using pre-defined hypotheses. Another strength of the study is that, to the best of the authors’ knowledge, it was the first study to measure the responsiveness on the index by determining the correlation of change scores between the NDI-U and the VAS_disability_.

## Conclusion

The NDI-U is a reliable, valid and responsive questionnaire that has a 2-factor structure. It consists of simple words that can be easily understood by the patients. Therefore, the NDI-U can be used to evaluate neck disability in Urdu-speaking patients with CMNP in clinical and research settings.

## Additional file

Additional file 1: Urdu version of the neck disability index. (JPG 3061 kb)

## Abbreviations

CMNP: Chronic mechanical neck pain
COSMIN: COnsensus-based Standards for the selection of health status Measurement INstruments
GROC: Global rating of change scale
ICC: Intra-class correlation coefficient
NDI: Neck disability index
NDI-U: Urdu version of the neck disability index
SD: Standard deviation
SDC: Smallest detectable change
SEM: Standard error of measurement
VAS_disability_: Visual analogue scale for disability
VAS_pain_: Visual analogue scale for pain.
