# Supplementary figures and images for: Urdu version of the neck disability index: a reliability and validity study
Source: BMC Musculoskelet Disord. 2017 Apr 8;18:149. doi: 10.1186/s12891-017-1469-5 (PMC5385030; doi:10.1186/s12891-017-1469-5)

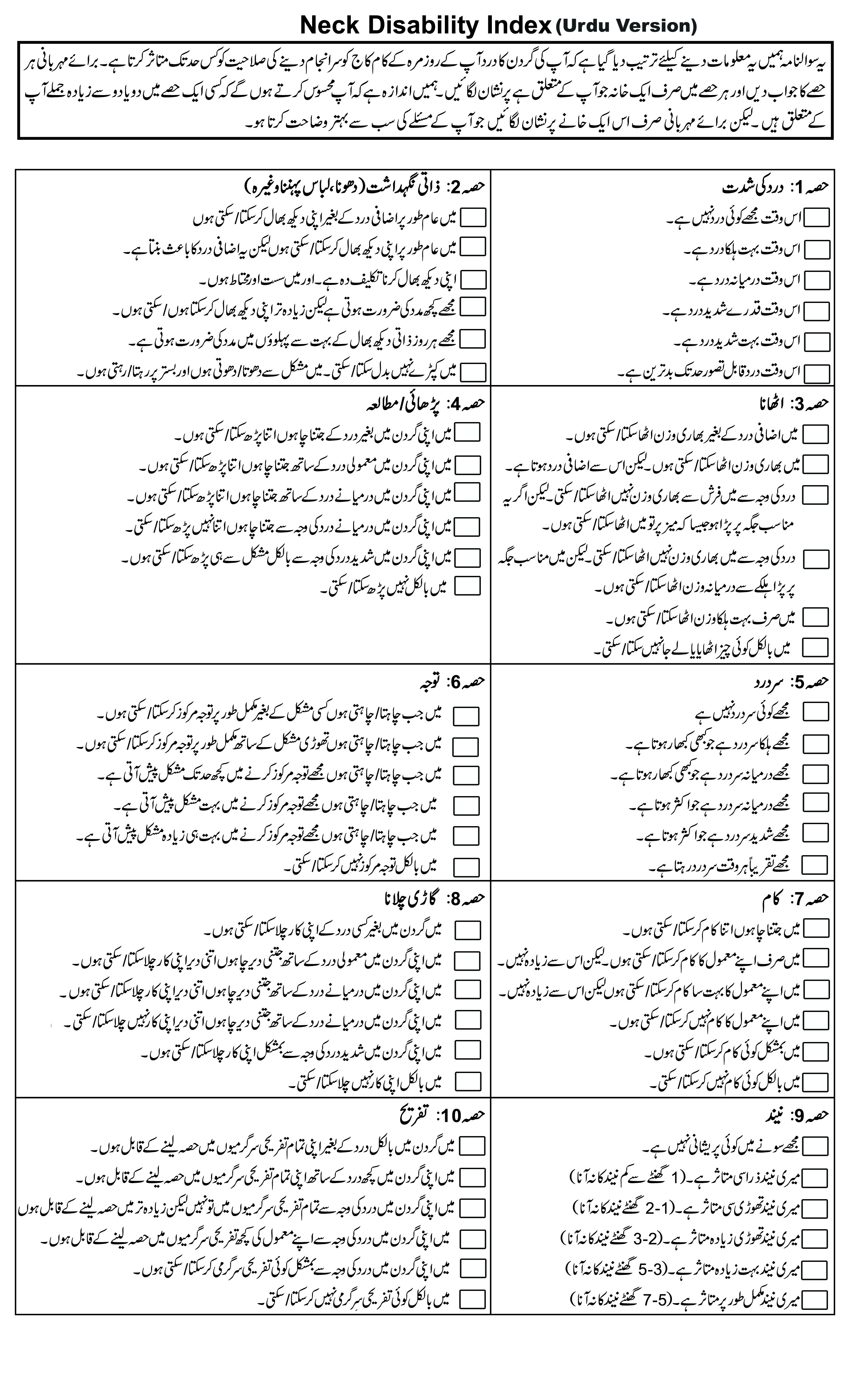

Supplement: Additional file 1: — Urdu version of the neck disability index. (JPG 3061 kb) [file 12891_2017_1469_MOESM1_ESM.jpg]
